# Developing the knowledge and library services researcher: A pilot project

**DOI:** 10.1111/hir.12579

**Published:** 2025-07-10

**Authors:** Gil Young, Maria J. Grant

**Affiliations:** ^1^ Workforce, Training and Education NHS England Manchester UK; ^2^ MJG Consulting Manchester UK

**Keywords:** continuing professional development (CPD), learning, librarians, health science, libraries, health care, library and information professionals, research capacity, research skills

## Abstract

NHS knowledge and library specialists support researchers and undertake research. The aim of this pilot programme, delivered as part of the Knowledge for Healthcare Learning Academy offer, was to equip learners from the health and social care library and knowledge sector in England with: the knowledge, skills and confidence to undertake research which adds to the evidence base for the profession; and/or equips them to contribute as a member of a healthcare research team.

## INTRODUCTION

Writing in 2008, Rossall et al. described the value of involving health librarians in the research activity of the NHS and the need to ensure they have the skills to contribute ‘*at an effective and productive level*’ (Rossall et al., 2008). This view was echoed by Kemp et al. (2019) who emphasis the value of ‘*having a positive research culture within any profession … particularly in healthcare*’. The Knowledge for Healthcare strategic framework (Health Education England, 2021a) further champions the value of NHS knowledge and library specialists in contributing to the work of healthcare research teams and in undertaking their own research.

A report commissioned by RLUK (Research Libraries UK) in partnership with AHRC (Arts and Humanities Research Council) identified several challenges to knowledge and library specialists being involved in research (Evidence Base, 2021). Among these were issues around perceptions of the role of knowledge and library specialists, capacity, and the expertise and knowledge required to undertake research.

Alongside the challenges identified above, it has been demonstrated that librarians often enjoy doing research, and such work directly improves both their own skills and practices for others. (Crampsie et al., 2020). Other identified benefits include:

At the individual level … a sense of accomplishment and contribution to the profession; increased understanding of and ability to participate in the research [of the organisation], career advancement and greater awareness of the latest library research … At an organisational level, librarians research and publication can help justify staffing, space and budget, apply best practice to services for patrons and raise the library's profile … For librarianship as whole, research and publication raise the profile of [knowledge and library specialists]. (Thielen & Stuit, 2021)

## DEVELOPING THE PROGRAMME

The aim of this pilot programme, delivered as part of the Knowledge for Healthcare Learning Academy offer, was to equip learners from the health and social care library and knowledge sector in England with the knowledge, skills and confidence to undertake research which either adds to the evidence base for the profession, and/or equips them to contribute as a member of a healthcare research team thus ‘*[creating] new knowledge and [advancing] evidence‐based practice*’ (Lessick et al., 2016). In reference to working as part of a research team the development of the skills and knowledge to place knowledge and library specialists on an equal footing with other team members is a key factor in enabling their successful participation (Lorenzetti & Rutherford, 2012).

The programme structure was based upon the one developed for the leadership programmes offered to healthcare knowledge and library staff across England by the Knowledge for Healthcare Learning Academy working in partnership with the NHS Leadership Academy. These leadership programmes had their origins in the CILIP Leadership Programme (Alcock, 2016).

The programme focused on developing those skills less directly related to participants' abilities as librarians, such as literature searching and critical appraisal, as research has shown that it is other aspects of research, including analysis of data and statistics, where librarians demonstrate a lack of confidence (Crampsie et al., 2020).

The programme was aligned to Section 9: Research, of the CILIP (2021) Professional Knowledge and Skills Base (PKSB). At the end of this programme, it was planned that learners should be able to:

- Plan and undertake a research project from start to finish.
- Define a robust research question.
- Develop a well‐designed research study.
- Write a clear and persuasive research proposal.
- Define core contributions that a knowledge and library specialist can make to a wider research bid and appropriate costings for these.
- Demonstrate an understanding of some of the key characteristics of both quantitative and qualitative research methods.
- Demonstrate an understanding of quantitative research methods including the collection of data and basic analysis.
- Present research findings in a clear and coherent manner.
- Effectively disseminate the outputs of their research to maximise impact.
- Demonstrate knowledge of professional responsibility, integrity, and ethical considerations.
- Reflect on their own progress as a learner and demonstrate independent learning ability to support their continued professional development.

## THE LEARNERS

Ten places were advertised on the course in early January 2023. The applications were screened by the project lead alongside members of the Health Education England Knowledge and Library Leadership team.

There were 12 applications, and all 10 places were filled. One individual dropped out before the course started and their place was offered to, and accepted by, one of the other applicants. The accepted applicants came from a wide geographical area covering most of England and represented a variety of knowledge and library service roles including library assistant, clinical librarian, and library manager.

Of the 10 final accepted applicants, 7 of them completed the course. Two of those who dropped out did so because they secured new roles outside of the NHS, and the third individual left for personal reasons. The seven individuals who completed the course all reported high satisfaction levels with the experience. They all attended and presented at the in‐person celebration event, which took place in London in January 2024. Certificates of completion were sent to all seven learners in March 2024.

## PRE‐COURSE PREPARATION

Prior to starting the course, learners were requested to familiarise themselves with the Knowledge for Healthcare Learning Academy Research Toolkit (Health Education England, 2021b). Each learner met with their assigned coach in advance of the first session to discuss their expectations around the programme. The learners were randomly matched to one other learner on the course and asked to arrange a virtual meeting before the first taught session to ensure that everyone had met at least one person on the course prior to the start of the pilot.

## TAUGHT SESSIONS

There were eight taught 2‐h sessions. These were delivered via MS Teams over a period of 10 months at roughly 1‐month intervals with a break across the summer. The break was included to make the course more attractive to part‐time staff and those with caring responsibilities. Additionally, the break gave learners some breathing space from new taught content to focus on data collection and collation for their projects. The later sessions included blocks of time set aside for individuals to work on their research projects. PowerPoint slides were provided to the learners in advance of the sessions.

The sessions were interactive and took the learners through all the stages of undertaking research. The following topics were covered:

- Introduction to research and the Knowledge for Healthcare Framework.
- Turning an idea into a research question.
- Reviewing the literature.
- Designing the study.
- Funding research.
- Writing research proposals.
- Obtaining ethical and Trust approval.
- Collecting and collating data.
- Analysing and interpreting data.
- Writing up research.
- Disseminating findings.
- Putting research findings into practice.

## COACHING, ACTION LEARNING SETS, AND 1‐2‐1 SESSIONS WITH TRAINER

Each learner was provided with a coach and invited to book 3 coaching sessions over the length of the course. Coaching is a task‐based approach to workplace learning and development that aims to focus on learning from previous experiences to improve how things are done currently (NHS Improving Quality, 2015). They were also invited to participate in two action learning sets, a supportive environment used to enable small groups to meet regularly to discuss, reflect, and find solutions to work‐related issues or to develop skills in a common area of interest (NHS Digital, 2019a). This combined approach was used to enable learners to experience different possible types of support when undertaking research with the aim of them understanding the differences between the approaches, where they might be best used, and which, if either, they preferred or felt worked best for them. Additionally, learners were offered a 1‐2‐1 session with the trainer to address content‐specific queries and to receive writing support. All the learners who completed the course participated in these activities.

The coaching sessions and action learning sets were provided by trained coaches and facilitators from the Knowledge and Library team working at Health Education England (now part of NHS England). Due to changes in team personnel, some of the learners were coached by two different individuals over the course of the pilot.

## MASTERCLASSES

Three masterclasses took place in the break between taught sessions on: Marking the Case for Getting Involved in Research; What Happens When Research Goes Wrong; and The Value and Impact of Online Networks for Learning and Sharing. These were delivered via MS Teams by Dr. Alison Brettle, Director of the Centre for Applied Health Research, School of Health and Society, University of Salford. The masterclasses were open to all knowledge and library staff working across healthcare in England.

It was not compulsory for learners to attend these masterclasses, but it was strongly recommended that they did so. The course learners did take up this opportunity. The only learners who did not attend all the masterclasses were those who had prior commitments such as leave.

## PROJECTS AND OUTPUTS

Learners undertook two small‐scale research projects: one as an individual and the other as a group. They have been encouraged to make the output of their research publicly available, and many presented their findings at the Health Libraries Group (2024) conference. Topics included health literacy, the link between knowledge and library services and NHS staff wellbeing, and e‐books. The two group projects were commissioned from colleagues at Health Education England. Individual projects were chosen by the learners.

## CELEBRATION EVENT

The celebration event took place in London on the 17 January 2024. All the learners who had completed the course attended. Each learner delivered two presentations. One outlining the findings from the group projects and one as an individual detailing their individual projects and research journeys. After the event had taken place, all the participants were issued with a certificate to confirm their participation in the project.

## EVALUATION

Evaluation of the pilot took place in February 2024. It consisted of two retrospectives, structured facilitated meetings at the end of the project to capture the knowledge before the project team disbands (NHS Digital, 2019b). One of these focused on the experiences of the learners, the other on those of the organisers, facilitators, and trainers. Feedback for the pilot was good. In the retrospective, the learners rated it as 8 out of 10 and above. They reported that their confidence had increased in both supporting others doing research and in their own skills. They liked the fact that there was something tangible to show at the end of the course, the network they had developed with the other learners and really enjoyed the taught sessions.

In terms of improving the course next time round, learners suggested taking a flipped classroom approach to content delivery, being clearer about how long some aspects of the projects might take at the start, including expectations of continuing with the research projects after the taught element of the course had been completed. Clarity on how the coaching and action learning sets related to taught content, and negotiating protected time with managers to undertake the research itself, were also raised. The issues around having time and organisational support to undertake research reflect identified barriers to knowledge and library staff becoming accomplished researchers (Kennedy et al., 2020).

## RECOMMENDATIONS AND NEXT STEPS

There are plans to run the programme again in 2026. The pre‐course information and the course structure, including the additional support offered in terms of coaching and action learning sets, will be revised, based upon the feedback obtained from the two retrospectives.

One of the key successes of the programme, as reported by the learners in the retrospect, was the development of a network of peer support for the individuals on the course. The value of such support has been identified as crucial in developing the research capability of knowledge and library specialists (Brown et al., 2015; Hoffmann et al., 2023; Kennedy et al., 2020). To continue this support for the course participants and to encourage participation in research across the wider healthcare knowledge and library sector, a new community of practice has been formed on FutureNHS (NHS England, 2024), a free‐to‐register cloud‐based collaboration platform enabling them to connect, share, and learn together. The NHS KLS Researchers Community of Practice is open to anyone working in the health or social care sectors of England. Individuals wishing to join the community of practice need to sign up to FutureNHS. Once this has been approved, they can search for the NHS KLS Researchers Community of Practice and request to join the workspace.

## Data Availability

Data sharing not applicable to this article as no datasets were generated or analysed during the current study.
